# The hypospadias classification affected the surgical outcomes of staged oral mucosa graft urethroplasty in hypospadias reoperation: An observational study

**DOI:** 10.1097/MD.0000000000008238

**Published:** 2017-11-27

**Authors:** Dachao Zheng, Shi Fu, Wenji Li, Minkai Xie, Jianhua Guo, Haijun Yao, Zhong Wang

**Affiliations:** Department of Urology, Shanghai Ninth People's Hospital, Shanghai JiaoTong University School of Medicine, Shanghai, China.

**Keywords:** classification, hypospadias, reoperation, staged graft urethroplasty

## Abstract

The staged graft urethroplasty is a recommended technique for repairing complex hypospadias. This retrospective study aimed to investigate the outcomes of this technique in hypospadias patients undergoing reoperation and to analyze the underlying contributing factors including age, meatus location, and graft and suture type.

We retrospectively analyzed 40 hypospadias patients undergoing reoperation who received a staged oral graft urethroplasty, including 15 buccal mucosal grafts and 25 lingual mucosal grafts. Median age at presentation was 18.5 years, and median follow-up was 17.5 months (range 8–30 months). The patients were classified according to their original meatus location.

Twenty-five complications developed in 12 of 40 (30%) cases, including 6 fistulas (15%), 7 infections (17.5%), 9 cases of glans dehiscence (22.5%), and 3 cases of stenosis (7.5%). There was no significant difference in the overall complication rates between prepuberty and postpuberty groups. In addition, no significant difference in complications was found between the 2 graft techniques. The complications were significantly higher in the original perineal type compared with the original penoscrotal type (7/10 vs 5/30, *P* = .0031). Seven patients who originally had perineal hypospadias developed multiple complications.

Based on this study, the staged graft urethroplasty is an effective technique in reoperative hypospadias repairs with reasonable complication risk. The hypospadias classification affects the surgical outcomes.

## 1 Introduction

Numerous surgical techniques have been developed to correct hypospadias but incidence of complications is still high. Several urethroplasty techniques have been demonstrated to be feasible ways for both primary operations and reoperations, such as tubularized incised plate (TIP) urethroplasty, Duckett urethroplasty and Thiersch-Duplay urethroplasty.^[1–3]^ However, failure of hypospadias repair is mostly associated with penile skin loss. In addition, there has been increasing agreement in the last 2 decades that the urethral plate (UP) should be preserved in hypospadias repairs.^[4]^ The free grafts provide extragenital tissue as neoplates in the reoperations of these failed cases. Although the 1-stage inlay graft technique has been reported as a good option to correct failed hypospadias repair,^[1]^ the staged technique is preferred by surgeons in the treatment of complicated cases because it provides neo-UPs and adequate tissue for urethroplasty.^[5]^

We attempted to correct failed hypospadias repairs with a 1-stage technique but obtained unsatisfactory results.^[2]^ Thus, a 2-stage graft procedure was performed as the ultimate solution for those who had undergone several failed urethroplasties, even in the cases of hypospadias cripples. The aim of this study was to evaluate the surgical outcomes of the staged graft urethroplasty using oral mucosal graft (OMG) for complex hypospadias after previous failed surgeries.

## 2 Methods

Between January 2007 and December 2011, 40 cases of previous failed hypospadias surgeries were included in this study. In this series, all patients had undergone one or more previous failed hypospadias repairs, had severe scars surrounding the original UP or no healthy foreskin, and had penile skin available for a flap procedure. Four patients had dysuria due to urethral strictures. The operations were performed by 2 highly qualified surgeons (Hai-Jun Yao and Zhong Wang). This study was approved by the ethics committee of Shanghai 9th People's Hospital, Shanghai JiaoTong University School of Medicine.

**Figure 1:**
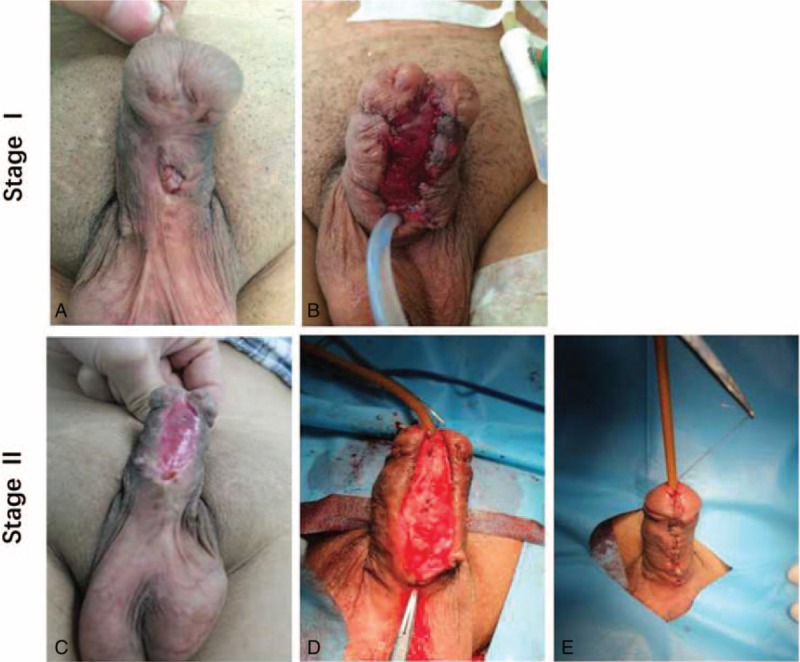
Surgery stages. The first stage (A, B) and the second stage (C, D, E).

In the first stage, all scar tissues were excised, including the scarred urethra (Fig. 1A). Penile straightening was tested by artificial erection. Dorsal plication was performed in 12 patients since chordee were noticed. Then, the glans wings were opened widely. The graft was harvested from the ventral surface of the tongue^[6]^ or the inner cheek^[7]^ according to surgeon's preference and patient's oral condition. The graft was quilted from the native plate to the glans to create a neo-UP (Fig. 1B). A protective tie overdressing was placed to reduce the chance of hematoma collecting under the graft.

The second stage was performed at least 6 months later using the standard Thiersch–Duplay technique (Fig. 1C). The neourethra was tabularized with 2-layer running subepithelial 6-0 absorbable PDS (Fig. 1D). Glansplasty was done with a 1-layer interrupted subepithelial 6-0 polyglactin (20 patients) or 6-0 absorbable PDS (20 patients) according to randomized selection (Fig. 1E). The catheter sizes were selected from 8 to 14 Fr, according to the size of the patient's penis. Scrotal dartos flap (15 patients) or tunica vaginalis flap (TVF; 25 patients) provided barrier flap coverage to all patients according to the patient's condition.

The urethral catheter was removed on postoperative day (POD) 7 after the first-stage and on POD 6 to 14 after the second stage. Antibiotics were administered intravenously until the catheters were removed. All patients accepted their follow-ups by a combination of outpatients and telephones.

Data were analyzed using the Fisher exact test because of a small sample size. A 2-side *P* value <.05 was considered statistically significant. All analyses were performed using SPSS (version 21.0, Chicago, IL).

## 3 Results

All surgeries went smoothly. The median age was 18.5 years. (2–45 years.). The urethral defect ranged from 3 to 7 cm, and the median length was 5 cm. The median number of prior operations was 2 (1–4 surgeries). No complications were observed in the first stage, except 3 patients with focal graft contracture requiring an additional operation to patch the graft in the second operation. The incision of the donor sites healed quickly within 5 days, and the tongues were completely recovered in appearance and function after 3 months without complications. The median duration of follow-up after the second stage was 17.5 months (range 11–30 months). Twenty-five complications (grade IIIb according to the Clavien Classification of Surgical Complications)^[8,9]^ developed in 12 of 40 (30%) individuals, including 6 fistulas (15%), 7 wound infections (17.5%), 9 cases of glans dehiscence (22.5%), and 3 cases of stenosis (7.5%). Patients with fistulas underwent repairs 6 months postoperatively and were cured with a single procedure. Three patients with stenosis were cured after 2- to 3-month repeated dilations. Three of the 9 patients with glans dehiscence underwent glansplasties and the rest of patients did not seek further repairs.

Several possible contributing factors to the complications were analyzed, including age at operation, type of graft, and original meatal location. In this study, patients were identified as prepubertal (0–10 years) or postpubertal (≥11 years) according to their age at the time of surgery following the Chinese pubescent classification criteria. There was no significant difference in the overall complication rates between these 2 cohorts (3/16 vs 9/24, Fisher exact test, *P* = .1311), except the incidence of dehiscence (1/16 vs 8/24, Fisher exact test, *P* = .0430). There was also no difference in the overall complication rates between the buccal mucosal graft (BMG) and lingual mucosal graft (LMG) (5/15 vs 7/25, Fisher exact test, *P* = .2584). There was no difference in the incidence of glans dehiscence, using 6-0 polyglaction or 6-0 absorbable PDS for glansplasty (2/20 vs 7/20, Fisher exact test, *P* = .0539). No statistically significant difference in complication rates was observed between the patients with 1 operation and the patients with more failed operations (3/15 vs 9/25, Fisher exact test, *P* = .1664). In this series, patients were classified as perineal type or penoscrotal type according to their original meatus locations. Thus, there were 30 penoscrotal hypospadias cases and 10 perineal hypospadias patients, respectively. The complications were significantly higher in the perineal type compared with the penoscrotal type (7/10 vs 5/30, Fisher exact test, *P* = .0031). All 7 patients developed multiple complications. In this study, 2 types of autogenous tissues (scrotal dartos flap and TVF) were transferred to cover the neourethra as waterproofing layers, but no difference of complication rate was observed (2/15 vs 4/25, Fisher exact test, *P* = .3460). Patient complications are summarized in Tables 1 and 2. Although several patients developed complications after operations, patients or their parents still admitted that there were big promotions of final cosmetic outcomes.

**Table 1:**
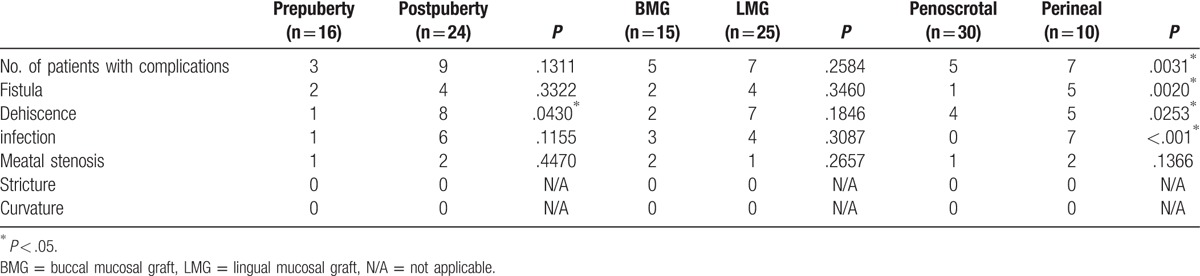
Complications between different ages, grafts, and classifications.

**Table 2:**
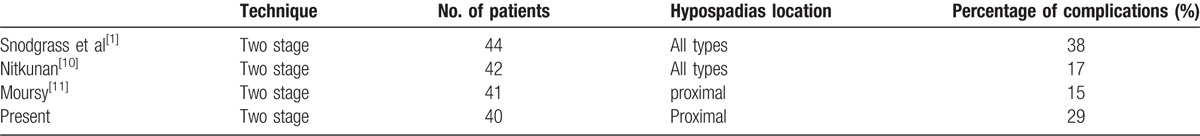
Comparing to the outcomes reported in other series.

## 4 Discussion

For most surgeons, 1-stage repair with skin flaps is a conventional solution for reoperative hypospadias urethroplasty. However, previous failures create skin paucity and severe scarring on the ventral penis, which lead to a high complication rate and unsatisfactory closure.^[12]^ Under this condition, the graft techniques provide extra tissue for the urethroplasty and achieve desirable results.^[13]^ The graft techniques are classified as single- and 2-stage approaches. The Snodgraft repair is a popular single-stage technique that achieves successful outcomes.^[14]^ In this procedure, dorsal midline TIP-like incision widens skin strip and graft is quilted into defect. However, decision making depends on quality of UP and foreskin. Poor quality of UPs in our patients indicated the Snodgraft technique was not feasible.

The 1-stage tubed graft is another single-stage technique. Grafts are rolled into tubes and then anastomosed to the original meatus. High breakdown rate of this technique was described, and lack of an adequately vascularized graft bed was believed as a contributing factor.^[15]^ Therefore, Goyal et al^[10]^ even recommended abandoning the 1-stage tubed OMG. Same disadvantage was observed in the 1-stage ventral onlay graft technique. On the contrary, the dorsal patching graft in the first stage can overcome this disadvantage and provide large, well-vascularized neoplates for severe hypospadias, which allow urethral tubularization without tension and also achieve desirable cosmetic and functional outcomes.^[13]^ Therefore, the staged graft technique is selected to repair these cases who lack normal UPs and adequate tissues for urethroplasty. This technique is considered a good alternative for those reoperative patients, including the repairs of posthypospadias strictures.^[16]^

Various substitute materials have been reported with satisfactory outcomes in the literature.^[15,17,18]^ A full-thickness skin graft was used initially in the Bracka procedure in 1995.^[17]^ Later, BMG was considered a better substitute because of its biological characteristics.^[18]^ The LMG urethroplasty was firstly reported in 2006 by Simonato et al.^[19]^ This graft can provide similar tissue characteristics of anti-infection, nonkeratinized epithelium as BMG. In addition, it is easier to be harvested. Thus, oral mucosal grafts including BMG and LMG were ideal choices for these patients. Because of the expected 20% shrinkage, the sizes of the harvested oral grafts are larger.^[20]^ In most pubertal patients, BMG was sufficient to be placed over the corpora cavernosa. LMG was more suitable in some adult patients with extremely huge long defects. Because each side of the ventrolateral aspect of the tongue can offer a maximum 7- to 8-cm-long and 1- to 2-cm-wide mucosal graft, 2 pieces of mucosa harvested from 2 sides can provide sufficient size for defects. Another advantage of LMG is that it is easier to be harvested than BMG.^[21]^

The complications were significantly higher in the perineal group compared with the penoscrotal group in our study, which indicated that the hypospadias classification (meatus location) affected the surgical outcome in the staged oral graft urethroplasty for the reoperative hypospadias. Seven cases of original perineal hypospadias were initially infected and then developed secondary complications. Secretions oozing from the wound were observed. On the contrary, there is only 1 patient developed stenosis and fistula in other 5 failed cases, whose original meatuses were located in penoscrotal junction. According to the results above, we speculate the wound infection may act as a trigger in the development of complications. At first, we considered age was the only factor facilitating infection since there were 6 postpubertal patients in the 7 original perineal cases. Later, we found the secretions were observed in most patients from the POD 3 to 5, but only these 7 perineal hypospadias patients were infected. This result suggested that the original meatus location may be a more important contributing factor than age. There were 2 potential reasons below. First, the scrotal skin fold was easy to hide grease and bacteria, which may lead to a high incidence of infection. Furthermore, the neourethra lacking of smooth muscle in the perineal was always lax and dilated. Once lower urinary tract symptoms caused by the stimulation of catheter appeared, urine remnant would easily happen and infection would develop soon.

In this clinical study, glans dehiscence was the most common complication. Several possible contributing factors have been discussed.^[11,22]^ Snodgrass et al^[23]^ suggested that the anatomical (proximal hypospadias) and/or host factors (wound healing) were major factors. These major factors may also present in our study. Therefore, age, type of suture, and some other factors should also be discussed. Penile erection was easier to be observed in postpubertal patients, which was generally considered as a potential factor leading to wounds dehiscence. More than half patients in this series were postpubertal males and the penile erection may, to some extent, affect the wound. In addition, Snodgrass et al^[1]^ suggested that thin grafts such as lip mucosa might result in a low rate of glans dehiscence. According to his conclusion, the thicker grafts (BMG and LMG) we placed in the glans may be another factor. Wrong decisions of glansplasty may also lead to glans dehiscence because of tensioned suture on small glans. Therefore, some surgeons did not extend the neourethra into a small glans to avoid glans dehiscence.^[24]^ Different suture types were compared in numerous papers and most of results indicated that the glans dehiscence was independent of the suture types, which was consistent with the findings in our study.^[23]^

Generally, fistula is the major complication in tubularized techniques.^[25]^ Several techniques such as spongioplasty and second-layer covering have demonstrated satisfactory outcomes, leading to a low fistula complication rate.^[22,26,27]^ In this study, spongioplasty was unavailable because spongiosa on both sides of the UP had been destroyed in previous operations. The lack of sufficient adjacent dartos around the neourethra also forced us to use a scrotal dartos flap or TVF as a waterproofing layer, which has been associated with a significant decrease in fistulas.^[28,29]^ Dhua et al^[30]^ concluded that TVF had an advantage over dartos fascia for soft tissue coverage of the neourethra. However, Chandrasekharam and Jayaram^[31]^ thought that TVF only had an advantage in reoperative hypospadias repairs in which dartos tissue was unavailable. The fistula rates of scrotal dartos flap and tunica vaginalis were not significantly different in our study. Our outcomes were similar to these reported data and demonstrated that a second-layer transferred from a scrotal dartos flap or TVF can achieve a low primary fistula incidence. Of the 6 fistulas in this series, 5 were secondary to the wound infection and 1 was a late fistula due to the meatal stenosis in POD 30, which had also been mentioned in Rompré et al's study.^[32]^

Similar to fistulas secondary to wound infections, some other complications were also secondary. For this reason, the complication rate was much higher than the operation failure rate. The final number of patients with complications was an acceptable result in this study, which was comparable to the outcomes reported in other studies (Table 2). Snodgrass et al,^[1]^ Nitkunan et al,^[18]^ and Moursy^[25]^ reported complication rates of 38%, 17%, and 15% using a 2-stage hypospadias repair technique with BMG. Although different grafts were used, the total complication rate was slightly higher. Simonato et al^[33]^ reported a final success rate of 0% with a 2-stage repair utilizing a hybrid BMG and LMG. However, this result was obtained from only 5 patients with failed hypospadias repair, and the small sample size may impair the validity of the results.

Patients, including those failed cases, experienced substantial improvements after operations, and they or their parents were satisfied with the postoperative cosmetic outcomes. From our perspective, although some of them had normal glans and a slit neomeatus, the appearance of the repaired penis was different from a normal penis. Their penises were always small, short, and swollen. We suspected that poor development of the penis was the major factor, and skin edema caused by multiple failed operations also led to a less-than-ideal appearance.

Our study was limited by a short follow-up period. Most problems occurred in the first postoperative year,^[20]^ but late complications were also observed in the long-term follow-ups, especially in patients after puberty. The curvature related to the rapid growth of the penis at that age is an example of a late complication. However, we still know little about the long-term behavior and growth of less androgen sensitive tissue such as oral mucosa. In addition, stricture, diverticula, hair growth, and uroliths often catch the attention of patients, but the urinary spray, stream deviation, and other unconventional late complications are always ignored by patients and urologists. Thus, a stable long-term follow-up is suggested for all patients. Another limitation of this study was its retrospective nature and a small number of included cases, which could lead to selection bias and affect the accuracy of statistical analysis.

## 5 Conclusions

In general, both LMG graft urethroplasty and BMG graft urethroplasty are remedial measures for failed cases, especially for patients who are short of healthy tissues for urethroplasty. When those perineal hypospadias patients need reoperations, urinary diversion is recommendable to prevent infection.
